# Awareness About Osteoporosis Among the General Population Based on the Osteoporosis Knowledge Assessment Tool (OKAT): A Cross-Sectional Study in the Northern Border Region of Saudi Arabia

**DOI:** 10.7759/cureus.56839

**Published:** 2024-03-24

**Authors:** Ekramy Elmorsy, Amgad N Elsawi, Nasser M Alruwaili, Abdulelah H Alruwaili, Sultan N Alanazi, Khalid R Alenezi

**Affiliations:** 1 Pathology, Northern Border University, Arar, SAU; 2 Anatomy, Northern Border University, Arar, SAU; 3 Surgery, Northern Border University, Arar, SAU

**Keywords:** osteoporosis knowledge assessment tool (okat), northern border region, bone density, pathological fractures, s: osteoporosis

## Abstract

Objective: Osteoporosis is a progressive systemic skeletal disease characterized by increasing susceptibility to fractures. The current study was conducted to assess the awareness about osteoporosis among the general population in the Northern Border region of Saudi Arabia to improve awareness and proper planning for public awareness about osteoporosis.

Methods: The study was conducted as a cross-sectional survey study, based on the online distribution of the Arabic-translated Osteoporosis Knowledge Assessment Tool (OKAT). The questionnaire questions cover the demographic characteristics of the participants, as well as symptoms, risk factors, prevention, and knowledge of treatment centers for osteoporosis in Saudi Arabia.

Results: 395 participants were enrolled in the study after their informed consenting. After scoring all correct answers for each participant, the mean score of all participants' answers was 12.5±3.4 (range 0-19). Participants with poor knowledge (0-7 scores), moderate knowledge (8-13 scores), and good knowledge (13-20 scores) represent 61 (15.4%), 213 (53.9%), and 121 (30.6%), respectively. The mean percentage of right answers to all the questions is 44.1%. The highest awareness level was shown in the area of osteoporosis symptoms and risk of fractures, while the lowest was recorded in the questions covering the risk factors. Ages, genders, jobs, and levels of education significantly affected the participants' levels of awareness.

Conclusion: The public awareness among the population in the Northern Border region about osteoporosis is less than satisfactory. More awareness activities targeting the risky groups should be planned especially in the area of risk factors and preventive measures for osteoporosis.

## Introduction

Osteoporosis is defined by the World Health Organization (WHO) as a bone mineral density (BMD) that is 2.5 standard deviations or more below the typical value for young, healthy women [1]. Low bone mass and the microarchitecture of deteriorating bone tissue increase the fragility and fracture risk of osteoporosis, a degenerative systemic skeletal disease. It is on par with diabetes and hypertension as a common chronic metabolic bone disease [2,3]. Osteoporosis is clinically significant when it leads to fractures, which typically happen in the distal radius, proximal humerus, pelvic, proximal femur, and vertebral bodies [4]. Globally, it is estimated that one in five males and more than one-third of adult women will experience one or more fragility fractures in their lifetime [1].

High morbidity is associated with osteoporosis-related fractures because of pain, incapacity, death, and financial load [3]. The two categories of osteoporosis risk factors are modifiable and non-modifiable. Non-modifiable risk factors include age, gender, ethnic origin, whether or not osteoporosis runs in the family, the date of the first menarche and menopause, and the history of fractures following minor trauma in first-degree relatives. The majority of modifiable factors include lifestyle choices like drinking alcohol or smoking, not getting enough exercise, and eating a diet low in calcium and vitamin D. Menopausal women are more susceptible to osteoporosis than other genders, resulting in a distinct condition known as postmenopausal osteoporosis [5-7].

Raising awareness about osteoporosis risk factors and prevention is essential, as evidenced by the general public's and healthcare stakeholders' persistent knowledge gaps in these areas [8-10]. For example, about 20% of patients in Switzerland had medical disorders that increased their risk of osteoporosis or were taking medication, according to a countrywide investigation. In addition, 3.5% of patients experienced fragility fractures, 7.3% of patients got treatment for osteoporosis, and 53.9% of patients did not use calcium or vitamin D supplements. Remarkably, only 38.5% of patients who responded to the previously published awareness survey acknowledged osteoporosis as a chronic illness [11]. Similar findings were found in a regional study conducted in Jordan on postmenopausal and premenopausal women; average scores for each group were 50.9 and 51.3 out of 100, respectively, indicating low knowledge regarding osteoporosis [12].

Another regional Egyptian study on premenopausal and postmenopausal women showed little knowledge of osteoporosis and fractures, as well as inadequate knowledge of diets high in calcium and vitamin D. Women who were not osteoporotic showed greater awareness [13]. Within Saudi Arabia, in 2016, a study was conducted to assess the general public's knowledge about osteoporosis among Saudi men and women. Just 44% of females showed satisfactory knowledge about the disease, compared to 58% of males. Additionally, those over 51 gave more accurate responses than those between the ages of 15 and 35 regarding osteoporosis [14]. Two other Saudi studies conducted in Riyadh revealed that Saudi women do not apply osteoporosis prevention methods with enough diligence [11,15]. A different study by Amarnath et al. [1] found that patients at Security Forces Hospital (SFH), Riyadh, Saudi Arabia, had a mean awareness score of 66% about osteoporosis. Comparing the other subscales of the Osteoporosis Knowledge Assessment Tool (OKAT) to the symptoms and fracture risk, as well as the preventative variables including diet and physical activity, the participants knew more. The knowledge of younger and older females (>40 years old) differed significantly, with younger females scoring lower on the OKAT.

As awareness about the medical problems is essential to follow the proper preventive and curative measures, and due to the shortage of data about awareness of the Saudi population in the Northern Border region regarding osteoporosis, the current study was conducted to assess the awareness of osteoporosis among the general population on the Northern Border region of Saudi Arabia to evaluate the current situation and assess the effect of the different demographic parameters on awareness level. Also, the study will supply the healthcare leader with the current status for proper planning for public awareness about osteoporosis.

## Materials and methods

The study was conducted as a cross-sectional survey with a convenient sampling technique, in the Northern Border region of Saudi Arabia. The Northern Border region is located in the country's north, bordering Iraq and Jordan. It has an area of 127 km^2^ and a population of 383,051 out of 34,218,169 of Saudi Arabia's population. It encompasses four main areas, Arar, Rafha, Turayf, and Al Uwayqilah, and many villages and towns. The inclusion criteria of the study considered only Saudi participants aged 18 years or more who reside in the Northern Border region of Saudi Arabia. The sample size was calculated using Epi Info. Taking a margin of error of 5%, the assuming proportion of 50%, a confidence interval of 95%, and a population of 383,051, the sample size will be 384.

The study design was evaluated and approved by the Northern Border University Bioethics Committee (approval number: 144/23/H). Informed consents were considered from participants, and confidentiality of data was considered in all the research project phases. An online self-administered questionnaire was utilized. The questionnaire was in Arabic and sent in Google Forms to be distributed through the widely used social media groups for the local Saudi persons in the Northern Border region. The questionnaire was divided into two sections: The first section assessed the demographic characteristics of the participants, such as age, gender, marital status, occupation, education, and nationality. The second section evaluated osteoporosis awareness, which adopted OKAT as a validated and reliable self-administered questionnaire, which is composed of 20 questions covering the different areas of awareness about osteoporosis including risk factors, bone fracture and symptoms, preventive measures, and treatment availability [16]. OKAT is a three-point Likert scale (True, False, I do not know). To ensure the face validity of the questionnaire translation, the survey was revised by family and community medicine department staff members. After their approval, a pilot study among 20 participants was conducted. The feedback from the pilot study was used to improve the clarity and comprehensibility of the questionnaire. Responses from the pilot study were not included in the final analysis. For evaluation of the level of awareness, true answers were considered as 1, while false answers and "I don't know" responses were considered as 0. Then the level of awareness was assessed by the sum of the scores.

Statistical analysis

Data was collected in an Excel sheet; inclusion and exclusion criteria were revised in the available participants' data to ensure that the enrolled participants were fulfilling the inclusion criteria. Descriptive statistics was used. The categorical variables were summarized as frequency and percentage, and the continuous variables were summarized as mean and standard deviation. A test of the association was used to evaluate the relationship between demographic characteristics and osteoporosis awareness. A p-value of <0.05 was considered statistically significant. All statistical analyses were performed using PRISM 5 (GraphPad Software Inc., San Diego, CA).

## Results

The current study was conducted to evaluate the level of awareness about osteoporosis among Saudi people in the Northern Border region of Saudi Arabia using OKAT. After consenting, 395 participants were enrolled in the study. Participants aged from 21 to 40 represented the majority of the participants (58.2%). Males represented 51.4%, while females represented 48.6%. Health professionals represented 18.7% of all persons enrolled in the study. The full demographic data of the participants are shown in Table 1.

**Table 1:** Demographic data of participants enrolled in the study

Parameter	Groups	Frequency	%
Age groups	<21	39	9.9
	21-40	230	58.2
	>40	126	31.9
Gender	Female	192	48.6
	Male	203	51.4
Job	Health professional	74	18.7
	Non-health professional	321	81.3
Education	Pre-university	70	17.7
	University	325	82.3
Total		395	100

After scoring all correct answers for each participant, the mean score of all participants' answers was 12.5±3.4 (range 0-19). According to the OKAT score, the participants were classified into subclasses, poor knowledge (0-7 scores), moderate knowledge (8-13 scores), and good knowledge (13-20 scores), representing 61 (15.4%), 213 (53.9%), and 121 (30.6%), respectively.

As shown in Table 2, the mean percentage of right answers to all the questions is 44.1%. The highest percent of correct answer was in question number 1 (93.7% right answers) which was regarding osteoporosis leading to increased risk of bone fracture, followed by question 12 (75% right answers) about the importance of milk and dairy products as a source of daily calcium supply. In contrast, the lowest levels were recorded in question numbers 2 and 16 considering bone pain as a manifestation of osteoporosis prior to fractures and bone mass loss after menopause (8.4% and 6.8% right answers, respectively).

**Table 2:** Participants' responses and awareness level for each OKAT questionnaire questions OKAT: Osteoporosis Knowledge Assessment Tool

Questions	Responses						Awareness score	
	True		False		I do not know			
	n	%	n	%	n	%	n	%
Osteoporosis increases the chance of bone fractures.	370	93.7	4	1.0	21	5.3	370	93.7
Osteoporosis typically manifests as symptoms (such as pain) prior to fractures.	300	75.9	33	8.4	62	15.7	33	8.4
The development of osteoporosis in old age is not prevented by having a higher peak bone mass at the end of childhood.	129	32.7	122	30.9	144	36.5	129	32.7
Men have osteoporosis more frequently.	96	24.3	165	41.8	134	33.9	165	41.8
Osteoporosis risk can be raised by cigarette smoking.	237	60.0	38	9.6	120	30.4	237	60.0
When compared to other races, white women have the highest fracture risk.	160	40.5	83	21.0	152	38.5	160	40.5
A fall is just as important as low bone strength in causing fractures	213	53.9	131	33.2	51	12.9	213	53.9
The majority of women have osteoporosis by the age of 80.	241	61.0	62	15.7	92	23.3	241	61.0
By the time they are 50 years old, the majority of women will have at least one fracture before they die.	122	30.9	145	36.7	128	32.4	122	30.9
Physical activity of any kind is beneficial for osteoporosis.	264	66.8	56	14.2	75	19.0	56	14.2
My clinical risk factors make it simple to determine whether I am at risk for osteoporosis.	213	53.9	30	7.6	152	38.5	213	53.9
A person is more likely to develop osteoporosis if they have a family history of the disease.	233	59.0	70	17.7	92	23.3	233	59.0
Two glasses of milk every day will provide enough amount of calcium.	298	75.4	37	9.4	60	15.2	298	75.4
For those who cannot consume dairy products, sardines and broccoli are good calcium sources.	252	63.8	23	5.8	120	30.4	252	63.8
Calcium pills alone can stop bone loss.	100	25.3	172	43.5	123	31.1	172	43.5
An osteoporosis risk factor is a high salt intake.	146	37.0	69	17.5	180	45.6	146	37.0
In the 10 years following the start of menopause, there is a slight loss of bone mass.	222	56.2	27	6.8	146	37.0	27	6.8
After menopause, hormone therapy stops additional bone loss at any age.	174	44.1	51	12.9	170	43.0	146	37.0
Alcohol intake increases the risk of osteoporosis.	159	40.3	138	34.9	98	24.8	159	40.3
The "Saudi" region lacks any efficient osteoporosis therapies.	210	53.2	52	13.2	133	33.7	152	38.5
The mean score of the correct answers							175.3	44.1

In the different areas of awareness covered by OKAT, the highest awareness level was shown in the area of osteoporosis symptoms and risk of fractures, while the lowest was recorded in the questions covering the risk factors (Table 3).

**Table 3:** Participants' responses to the four different areas covered by OKAT questionnaire questions OKAT: Osteoporosis Knowledge Assessment Tool

Awareness area	Number of questions	Expected total score	Actual minimum score	Actual highest score	Actual score mean±SD (%)
Osteoporosis risk factors	8	8	0	7	3.7±2.1 (53%)
Pain and fracture risk	5	5	0	4	2.7±1.8 (54%)
Preventive factors	5	5	0	5	2.4±2.4 (48%)
Treatment availability	2	2	0	2	0.8±0.7 (40%)

The chi-squared test was used to examine the difference between the categorical variables of the demographic data to assess their effect on the awareness levels among the participants. Data revealed that ages, genders, jobs, and levels of education significantly (p<0.0001) affected the participants' levels of awareness. More numbers of participants scored above 13 (high knowledge score) among participants in the age group between 21 and 40 years, female participants, healthcare professionals, and university-educated individuals in comparison to the other groups of participants related to the same parameters (Table 4).

**Table 4:** Effect of demographic data on the participant estimated knowledge scores based on their responses to OKAT questionnaire questions OKAT: Osteoporosis Knowledge Assessment Tool

Parameters	Groups	Poor knowledge score (0-7)		Intermediate knowledge score (8-13)		High knowledge score (14-20)		Total		Chi-squared test
		n	%	n	%	n	%	n	%	P<0.0001*** df=34.29, 4
Ages	<21	12	30.8	15	38.5	12	30.8	39	100	
	21-40	32	13.9	132	57.4	66	28.7	230	100	
	>40	17	13.5	66	52.4	43	34.1	126	100	
Gender	Female	23	12.0	86	44.8	83	43.2	192	100	P<0.0001*** df=28.03, 2
	Male	38	18.7	127	62.6	38	18.7	203	100	
Jobs	Health professional	19	25.7	21	28.4	34	45.9	74	100	P<0.0001*** df=24.16, 2
	Non-health professional	42	13.1	192	59.8	87	27.1	321	100	
Education	Pre-university	32	45.7	27	38.6	11	15.7	70	100	P<0.0001*** df=60.38, 2
	University	29	8.9	186	57.2	110	33.8	325	100	

## Discussion

The current study was conducted to evaluate the level of awareness in the Northern Border region of Saudi Arabia about osteoporosis using the well-established OKAT as an assessment tool which covers knowledge about osteoporosis symptoms, risk factors, preventive measures, as well as treatment availability. A survey was distributed to the participants, which included the OKAT and demographic characteristics. The study revealed that the mean score of all participants' answers was 12.5±3.4 (range 0-19). Participants with poor knowledge (0-7 scores), moderate knowledge (8-13 scores), and good knowledge (13-20 scores) represent 61 (15.4%), 213 (53.9%), and 121 (30.6%), respectively.

The estimated mean score of the correct answers was about 44.1%, which is lower than the previously published Saudi data based on the OKAT survey from the other areas of the Kingdom. According to Alghamdi et al. [2], the 376 study participants in our analysis had a mean score of 66% for accurate answers. A 2019 study [17] that included students from four different universities in Saudi Arabia and spanned 18-30 years found that the mean score for the total level of knowledge was 55.7% for females. A study conducted on 390 adult female participants in Majmaah City, Saudi Arabia, revealed that half of the participants (50%) knew a reasonable amount about osteoporosis [18]. Regionally, Qatari women of reproductive age scored 61.4% overall in knowledge [19]. This score was comparable to that of Turkish and Swedish women, who had corresponding scores of 63% and 61% [19,20]. This variation in the values of the mean score is explained by the different sampling groups and their demographic data affecting the outcome of the study.

It is essential to ascertain the degree of information that the various populations possess regarding the risk factors, since this knowledge can serve as a roadmap for creating osteoporosis preventive initiatives [21]. According to the current study, the real mean score for answering questions on risk factors was 52.8%, falling within the range of previously released data that indicated 54.5% of the study subjects were aware of the risk factors. Menopause, age in women, smoking, and family history were all recognized as risk factors for osteoporosis by nearly 60% of participants [2]. The current research's degree of family history awareness is higher than that of Alghamdi et al.'s [2] study, which found that 56.1% of respondents were aware of it, and another study carried out at a primary healthcare center in Dirab, Riyadh, Saudi Arabia, where only 22% of the females recognized family history as a contributing factor [22]. Participants in Qatar scored lower (36.%) than we did [20]. Of the 622 women surveyed in New Zealand in 2007, only 22% were aware that they were more likely to develop osteoporosis if their family had a history of the condition [23]. Another risk factor listed in the questionnaire was smoking. In other studies, smoking was found to be a risk factor for osteoporosis at different levels (58.1% and 67.8%) [2,24]. A 2018 study on postmenopausal women in Lebanon found that just 36% of them were aware of the dangers of smoking [13]. In a different Pakistani study, only 15% of participants were aware that smoking can cause the illness [23].

Comprehending the gravity of osteoporosis is vital in order to alter specific health-related practices linked to nutrition and exercise. Among the preventive variables included in the survey was an appropriate intake of calcium. It's critical to consume enough calcium each day to lower the risk of osteoporosis [24]. Of the participants, 75% knew the necessary daily amount of calcium, and about 65% recognized broccoli and sardines as calcium-rich foods. The fact that our study's percentages were greater than the study conducted on Saudi female college students, whose scores were 68% and 57%, respectively, may have something to do with the age group in which our study was conducted [25]. Just over half of the participants in the previously cited New Zealand study recognized broccoli as a calcium source [23], while only a small percentage of Qatari women in another study were aware that sardines and broccoli are high in calcium [12]. This highlights how crucial it is to understand the non-dairy sources of calcium, particularly for those on a vegan diet or who are lactose intolerant.

Determining whether there are knowledge gaps between younger and older females was one of our goals. The age and knowledge level were found to be statistically significantly correlated in this study. In all four of the OKAT subscales, females over 40 scored higher. Similar findings were found in a nationwide cross-sectional study conducted in Saudi Arabia in 2016 [14], particularly in the 51-65-year age range. In a similar vein, a 2006 study conducted in Pakistan [26] found that younger women knew less than older women. Conversely, a 2007 study on the Greek population [27] revealed that older ladies lacked adequate awareness regarding osteoporosis.

The current study's cross-sectional design with a statistically sufficient sample ensures data robustness and generalization. Also, the study used a widely accepted validated questionnaire which gives a satisfactory level of the collected data robustness for analysis and interpretation. However, its convenience sampling, selecting only the easily available participants group through social media, contributed to one of its limitations. Larger populations from various areas and Saudi regions should be included in future studies. The population's awareness of osteoporosis symptoms and the advantages of physical activity should also be investigated further, as these topics received the lowest marks for accurate responses.

## Conclusions

Awareness about osteoporosis is crucial for the proper diagnosis and management of the cases. The current data based on OKAT revealed an unsatisfactory level of awareness among the population in the Northern Border region of Saudi Arabia specially in areas of risk factors and preventive measures which are essential for the prevention and proper management of the cases through follow-up and proper seeking of the medical advice among the high-risk persons. Data is going in accordance with the previous data collected in Saudi Arabia. This means further planning for public awareness should be implemented especially among the high-risk group population at both the national and local level in the Northern Border region.

## References

[1] Amarnath SS, Kumar V, Das SL (2023). Classification of osteoporosis. Indian J Orthop.

[2] Alghamdi A, Almutairi OA, Abu Alqam R, Jambi A, Alharthi HS, Binhamran K, Mosli H (2023). Evaluation of osteoporosis perception among Saudi Arabian premenopausal women: a cross-sectional survey study using the Osteoporosis Knowledge Assessment Tool (OKAT). Cureus.

[3] Alqahtani GM, Alghamdi AM (2021). Assessment of osteoporosis knowledge among adult Saudi females attending the family medicine department at Security Forces Hospital, Riyadh, Saudi Arabia. J Family Med Prim Care.

[4] Mousavibaygei SR, Bisadi A, ZareSakhvidi F (2023). Outdoor air pollution exposure, bone mineral density, osteoporosis, and osteoporotic fractures: a systematic review and meta-analysis. Sci Total Environ.

[5] Walker MD, Shane E (2023). Postmenopausal osteoporosis. N Engl J Med.

[6] Zhu Z, Liu M, Zhang Y (2023). Risk factors for the comorbidity of osteoporosis/osteopenia and kidney stones: a cross-sectional study. Arch Osteoporos.

[7] Azeez TA (2023). Osteoporosis and cardiovascular disease: a review. Mol Biol Rep.

[8] Alfadhul SA, Abbas ZH (2023). Assessment of knowledge and beliefs toward osteoporosis among Iraqi perimenopausal women. Al-Rafidain J Med Sci.

[9] Saltık H, Öztürk F, Emiroğlu C, Hekimoğlu B, Aypak C (2023). Knowledge, attitude, and behavior levels of postmenopausal women about osteoporosis. J Bone Metab.

[10] Mahmoud Ahmed A, ELsayed Mahdy N, Fathy Mahmoud S (2024). Assessment of knowledge, health belief, and self efficacy for patients with osteoporosis. Egypt J Health Care.

[11] Barzanji AT, Alamri FA, Mohamed AG (2013). Osteoporosis: a study of knowledge, attitude and practice among adults in Riyadh, Saudi Arabia. J Community Health.

[12] Al-Muraikhi H, Said H, Selim N, Chehab MA (2017). The knowledge of osteoporosis risk factors and preventive practices among women of reproductive age in the state of Qatar: a cross-sectional survey. Int J Community Med Public Health.

[13] Ahmadieh H, Basho A, Chehade A, Al Mallah A, Dakour A (2018). Perception of peri-menopausal and postmenopausal Lebanese women on osteoporosis: a cross-sectional study. J Clin Transl Endocrinol.

[14] Tlt AE, Barghash SS, Al-Salamah NI (2016). Knowledge, attitude and practice (KAP) regarding osteoporosis among general population in Saudi Arabia. J Adv Med Med Res.

[15] Alshammari KF (2014). Women knowledge, attitude and practices about osteoporosis prevention "Riyadh Saudi Arabia". World J Med Sci Community Heal Nurs Ment Heal Dep.

[16] Winzenberg TM, Oldenburg B, Frendin S, Jones G (2003). The design of a valid and reliable questionnaire to measure osteoporosis knowledge in women: the Osteoporosis Knowledge Assessment Tool (OKAT). BMC Musculoskelet Disord.

[17] Khan JA, McGuigan FE, Akesson KE, Ahmed YM, Abdu F, Rajab H, Albaik M (2019). Osteoporosis knowledge and awareness among university students in Saudi Arabia. Arch Osteoporos.

[18] ElTohami K, Sami W, Eidan A, Mubarak M, Alotaibi F (2015). Study of knowledge, attitude and practice of osteoporosis among adult women in Majmaah City, Saudi Arabia. Int J Heal Rehabil Sci.

[19] Waller J, Eriksson O, Foldevi M (2002). Knowledge of osteoporosis in a Swedish municipality--a prospective study. Prev Med.

[20] Ungan M, Tümer M (2001). Turkish women's knowledge of osteoporosis. Fam Pract.

[21] Riaz M, Abid N, Patel J, Tariq M, Khan MS, Zuberi L (2008). Knowledge about osteoporosis among healthy women attending a tertiary care hospital. J Pak Med Assoc.

[22] Al-Shahrani FM, Al-Zahrani AM, Al-Haqawi AI (2010). Knowledge of osteoporosis in middle-aged and elderly women. Saudi Med J.

[23] von Hurst PR, Wham CA (2007). Attitudes and knowledge about osteoporosis risk prevention: a survey of New Zealand women. Public Health Nutr.

[24] Alshareef SH, Alwehaibi A, Alzahrani A, Fagihi A, Alkenani A, Alfentoukh M (2018). Knowledge and awareness about risk factors of osteoporosis among young college women at a university in Riyadh, KSA. J Bone Res.

[25] Bilal M, Haseeb A, Merchant AZ (2017). Knowledge, beliefs and practices regarding osteoporosis among female medical school entrants in Pakistan. Asia Pac Fam Med.

[26] Borer KT (2005). Physical activity in the prevention and amelioration of osteoporosis in women : interaction of mechanical, hormonal and dietary factors. Sports Med.

[27] Alexandraki KI, Syriou V, Ziakas PD (2008). The knowledge of osteoporosis risk factors in a Greek female population. Maturitas.

